# Thoracic epidural anesthesia time-dependently modulates pulmonary endothelial dysfunction in septic rats

**DOI:** 10.1186/cc7950

**Published:** 2009-07-06

**Authors:** Stefan Lauer, Hendrik Freise, Martin Westphal, Alexander Zarbock, Manfred Fobker, Hugo K Van Aken, Andreas W Sielenkämper, Lars G Fischer

**Affiliations:** 1 Department of Anesthesiology and Intensive Care Medicine, University Hospital Muenster, Albert-Schweitzer-Str. 33, 48149 Muenster, Germany; 2 Center for Laboratory Medicine, University Hospital of Muenster, Albert-Schweitzer-Str. 33, 48149 Muenster, Germany

## Abstract

**Introduction:**

Increasing evidence indicates that epidural anesthesia improves postoperative pulmonary function. The underlying mechanisms, however, remain to be determined. Because pulmonary nitric oxide has been identified to play a critical role in pulmonary dysfunction in sepsis, we hypothesized that thoracic epidural anesthesia (TEA) modulates endothelial dysfunction via a nitric oxide-dependent pathway.

**Methods:**

Thirty-six Sprague-Dawley rats underwent sham laparotomy or induction of peritoneal sepsis caused by cecal ligation and puncture (CLP). Septic animals were then treated with either bupivacaine 0.5% or normal saline epidurally (15 μl/h^-1^) for 6 hours or 24 hours after injury. Previous experiments demonstrated that these time points correspond with a hyperdynamic (at 6 hours) and hypodynamic circulation (at 24 hours), respectively. In addition, two sham control groups received either bupivacaine 0.5% or normal saline epidurally (15 μl/h^-1^). Six and 24 hours after injury, hemodynamic measurements and arterial blood gas analyses were performed in awake, spontaneously breathing rats. Exhaled nitric oxide, bradykinin-induced pulmonary vasoconstriction (a surrogate marker of endothelial dysfunction), pulmonary wet/dry-weight ratio (an estimate of pulmonary edema), and myeloperoxidase activity (MPO, a surrogate marker of neutrophil infiltration into lung tisssue) were investigated at 6 and 24 hours by using an established model of isolated and perfused lungs.

**Results:**

In hyperdynamic sepsis, treatment with TEA resulted in reduced bradykinin-induced pulmonary vasoconstriction (*P *< 0.05) and lower levels of exhaled NO as compared with those in untreated septic rats (*P *< 0.05). However, the development of pulmonary edema or MPO activity in the lungs was not alleviated by sympathetic blockade in this phase of sepsis. Conversely, TEA led to an increased bradykinin-induced pulmonary vasoconstriction and pulmonary edema despite reduced exNO levels and pulmonary MPO activity in hypodynamic sepsis (each *P *< 0.05 versus CLP 24 h). Pulmonary gas exchange was only marginally affected under the influence of TEA in hypodynamic sepsis. Mean arterial pressure and heart rate were not affected beyond the changes caused by sepsis itself.

**Conclusions:**

The results of the present study suggest that TEA modulates the NO pathway and exerts positive effects on pulmonary endothelial integrity only in hyperdynamic sepsis. Whether the negative effects on endothelial function in hypodynamic sepsis have an impact on overall morbidity and mortality remains to be determined in future studies.

## Introduction

Thoracic epidural analgesia (TEA) is one of the most versatile and widely used neuronal deafferentation techniques to reduce effectively intraoperative sympathetic stimulation resulting from surgical trauma [1]. TEA is therefore an integral part of a multimodal concept to accelerate rehabilitation after major abdominal and thoracic surgery and is increasingly incorporated into therapeutic strategies in critical care medicine [2-4]. Whether TEA is also useful to accelerate the recovery of high-risk patients with severe sepsis and septic shock is still not fully understood.

In severe sepsis, pulmonary endothelial dysfunction plays a pivotal role in the development of respiratory failure [5]. In this context, overproduction of nitric oxide (NO) in the lungs has been identified as a major cause for the emergence of endothelial dysfunction and pulmonary edema [6,7]. Likewise, increasing evidence suggests that TEA attenuates pulmonary endothelial injury in ventilated patients after major abdominal surgery. A recently published meta-analysis outlined that TEA reduced the rate of respiratory failure and the duration of required ventilation in those patients [8].

We hypothesized that TEA modulates pulmonary endothelial dysfunction in sepsis *via *an NO-dependent pathway. The current study was therefore conducted as a prospective, randomized, controlled laboratory experiment to clarify whether and how TEA affects the pulmonary endothelial dysfunction typically seen in the presence of sepsis. Because the early phase of hyperdynamic circulation is typically followed by a late hypodynamic circulatory state in rats with sepsis secondary to cecal ligation and puncture (CLP) [9], another objective of this study was to determine the effects of TEA in the different phases of sepsis. These aims were investigated by using an established model of isolated and perfused rat lungs [10,11].

## Materials and methods

After approval of the District Government, 36 male Sprague-Dawley rats (Harlan Winkelmann, Borchen, Germany) weighing between 250 and 300 g were kept in a 12-hour light/dark cycle and had free access to food and water. The animals were included in the study after a 1-week acclimatization period.

### Animal preparation

Anesthesia was induced and maintained by isoflurane inhalation (minimum alveolar concentration (MAC) 1.4 Vol. %). During instrumentation, spontaneous respiration was maintained. Central venous and arterial lines (0.96 mm OD) (Liquiscan, Ueberlingen, Germany) were introduced via the right external jugular vein and the left carotid artery, respectively. Epidural catheters (0.61 mm OD) were inserted at the levels of L3/L4 and advanced to T6. A repeated negative liquid-aspiration test excluded subdural position of the catheter tip. All catheters were protected by a swivel device. After completion of the experimental protocol, the position of the catheter was verified in each animal by autopsy. This model of continuous thoracic epidural analgesia in permanently instrumented awake rats has been shown consistently to produce stable thoracic sympathetic blockade while maintaining hemodynamic stability [12-14].

A midline laparotomy incision was made, and the cecum was ligated just distal to the ileocecal valve so that continuity was preserved. The cecum was then punctured twice with an 18-gauge needle. Thereafter, the bowel was returned to the peritoneal cavity, and the wound was closed in two layers. Rats were then allowed to awaken. Fluid resuscitation with NaCl 0.9% (6 ml/kg^-1^/h^-1^) was started postoperatively and continued throughout the *in vivo *experiment until the aorta was ligated for the isolation and perfusion of the lungs. Fentanyl (Janssen Cilag GmbH, Neuss, Germany; 2 μg/100^-1 ^g/h^-1^) was continuously infused to ensure adequate analgesia [10].

### Experimental protocol

Because previous experiments demonstrated that CLP sepsis corresponds with a hyperdynamic (2 to 10 hours after CLP) and hypodynamic circulation (later than 18 hours after CLP) with a transition from a hyperdynamic to a hypodynamic circulation between 10 and 20 hours, the effects of TEA on pulmonary endothelial dysfunction were determined at 6 and 24 hours, respectively, after CLP [15,16]. Taking into consideration that endothelial integrity did not differ between 6 and 24 hours in sham animals in previous studies [17], sham-operated animals were investigated at 24 hours only.

After the instrumentation, rats were randomly divided into six groups (n = 6 each) by using a sealed and numbered envelope system. To ensure that the investigator was blinded to the study-group assignment, a technical assistant conducted the laparotomy procedure and the preparation of the epidural infusion. The SHAM group received a sham laparotomy followed by continuous TEA with normal saline (15 μl/h^-1 ^for 24 hours, SHAM); the SHAM + TEA group underwent sham laparotomy followed by continuous TEA with isobaric bupivacaine 0.5% (15 μl/h^-1 ^for 24 hours, SHAM + TEA). In the hyperdynamic sepsis group (6 hours after injury), normal saline (15 μl/h^-1^) was continuously administered epidurally for 6 hours after induction of CLP sepsis (CLP 6 h). The TEA-treated hyperdynamic sepsis group received CLP + continuous TEA with isobaric bupivacaine 0.5% (15 μl/h^-1^) for 6 hours (CLP 6 h + TEA). In the hypodynamic sepsis group, normal saline (15 μl/h^-1^) was continuously infused through the epidural catheter for 24 hours (CLP 24 h). The TEA-treated hypodynamic sepsis group received a continuous epidural infusion of isobaric bupivacaine 0.5% (15 μl/h^-1^) for 24 hours after injury (CLP 24 h + TEA). Concentrations of bupivacaine were used as described previously [10,13].

Six and 24 hours after sham laparotomy or induction of peritonitis, respectively, mean arterial pressure (MAP), heart rate (HR), and respiratory rate were measured, and blood was withdrawn from the arterial catheter in awake, spontaneously breathing rats. Arterial hemoglobin, hematocrit, pH, paO_2_, paCO_2_, HCO_3_^-^, actual base excess (ABE), leukocytes, and arterial serum lactate concentrations were determined with blood gas analysis (ABL, 620 Radiometer, Copenhagen, Denmark).

### Isolated perfused lungs

After hemodynamic measurements and blood gas analysis, rats were again anesthetized with intraperitoneally injected α-chloralose (50 mg/kg^-1^; Sigma Chemicals, Deisenhofen, Germany) and urethane (650 mg/kg^-1^, Sigma Chemicals) and were then tracheostomized with a 17-gauge cannula. The lungs were mechanically ventilated with warmed (35°C) and humidified 21% O_2_, 5% CO_2_, and balanced N_2 _by using a rodent ventilator (tidal volume, 1 ml/100 g^-1^; frequency, 60 breaths/min^-1^; Harvard Apparatus GmbH, Deisenhofen, Germany). End-expiratory pressure was set at 1 mm Hg.

For isolated lung perfusion, a sternotomy was conducted, followed by excision of sections of the right and left anterior chest wall to expose heart and lungs. After heparinization (100 U), rats were partly exsanguinated by needle aspiration (5 to 6 ml). A steel cannula (13-gauge) connected to the perfusion system was inserted through the pulmonic valve into the main pulmonary artery by incision in the right ventricle. A suture tied around the pulmonary artery and aorta secured the cannula. The latter prevented systemic blood flow. The circle was closed by a cannula (3.5 mm outside diameter) that was inserted through the apex of the left ventricle and secured with umbilical tape. The isolation and perfusion procedure strictly followed an established and standardized technique [10,17].

**Figure 1:**
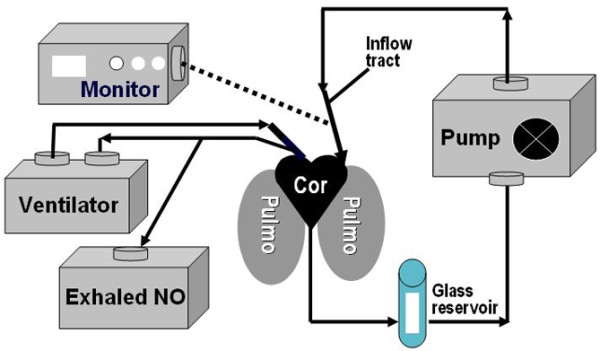
Illustration of the isolated and perfused lung model. After anesthesia and tracheostomy, exhaled NO (exNO) from rat's lungs was measured, followed by isolation and perfusion of the pulmonary circuit. Angiotensin II (Ang II) was injected into the inflow tract of the circuit to test vasoreactivity. Changes in perfusion pressure (Δp) from baseline were measured in millimeters of mercury. After a stabilization phase, bradykinin (BK)-induced pulmonary vasoconstriction was induced. Therefore, increasing concentrations of BK (1, 3, and 6 μg) were injected into the inflow tract. Changes in perfusion pressure were measured in mm Hg and expressed as Δp from baseline pressure.

The perfusate consisted of the rat's own blood (added to the perfusate after lung isolation) diluted with physiologic salt solution (containing in mmol/L: 119.0 NaCl, 4.7 KCl, 1.17 MgSO_4 _× 7 H_2_O, 22.6 NaHCO_3_, 1.18 KH_2_PO_4_, 3.2 × 2 H_2_O, 5.6 dextrose) to a hematocrit of 9% to 12%. Indomethacin (30 μg/ml^-1^) was added to block prostaglandin synthesis. The perfusate drained from the left ventricle to a glass reservoir and was heated to 38°C by a circumferential water jacket. With a peristaltic pump (Harvard Apparatus), the perfusate was returned to the pulmonary artery at constant flow (16 ml/min^-1^). The isolated lung preparation remained in the thoracic cavity lying supine and was warmed with a heating lamp. A warmed and humidified chamber was placed over the thoracic cavity to maintain thoracic temperature at 37°C. Reservoir pH was continuously monitored (Hanna Instruments) and maintained at 7.35 to 7.45 by addition of HCl or NaOH, as required. Pulmonary artery pressure (P_a_) was continuously monitored by using a pressure transducer (Becton Dickinson, Singapore). Mean pulmonary venous pressure was set at 2 mm Hg by adjusting the height of the reservoir and was held constant. Normoxic (21% O_2_) and hypoxic (3% O_2_) gas mixtures were administered through individual flowmeters. The inspired O_2 _concentration was monitored (Oxydig, Draeger, Germany) near the tracheal tube. The model of the isolated and perfused lungs is illustrated in Figure 1. After thoracotomy and stabilization of isolation and perfusion of the lung, receptor-mediated vasoconstriction was studied, as described later.

### Exhaled NO

Exhaled NO (exNO) was used as a surrogate marker for pulmonary NO production [18]. Before the isolation and perfusion of the lungs, the exhaled air from the rats' lungs was collected in a bag for 10 minutes. The exNO concentration was analyzed with a TE 42S NO_x _analyzer (Thermo Environment, Franklin, MA, USA) by using an established protocol [10].

### Receptor-dependent pulmonary vasoconstriction

Integrity of vascular smooth muscle cell (VSMC) function is critical for the regulation of the vascular tone. In this study, the potent receptor-mediated vasoconstrictor angiotensin II (Ang II, 0.1 μg; Sigma Chemicals) was injected into the inflow tract of the isolated perfused lung circuit to test possible functional impairment of VSMCs caused by sympathetic blockade or sepsis. Changes in perfusion pressure were measured in millimeters of mercury and expressed as pressure difference (Δp) from baseline pressure [10,17,19].

To test pulmonary endothelial dysfunction, bradykinin (BK)-induced vasoconstriction was evaluated by injecting increasing concentrations of BK (1, 3, and 6 μg; Sigma Chemicals) into the inflow circuit. Each dose was administered 5 minutes after the perfusion pressure returned to baseline levels. Changes in perfusion pressure were measured in millimetres of mercury and expressed as Δp from baseline pressure [11,17,20].

### Myeloperoxidase (MPO) activity

After the experiment, right lungs were immediately frozen and stored at -70°C until use. MPO, indicating pulmonary neutrophil infiltration, was measured in equal-sized samples of the right lungs by using an established protocol [21]. Results were expressed as microunits of MPO per milligram of protein of supernatant, as determined by bicinchoninic acid assay (Pierce Chemical Co., Rockford, IL, USA).

### Pulmonary wet- to dry-weight ratio

Pulmonary bloodless wet- to dry-weight ratio was determined at the end of the experiment. The left bronchus was carefully separated from the right bronchus and tied, and the left lung was severed. The lung was weighted immediately and dried at 60°C in an oven for 4 days to determine the ratio of wet-lung weight (WW) to dry-lung weight (DW). The dry-lung weight became stable after 48 hours of desiccation in our study [10].

### Statistical analysis

Sigma Stat. 3.1 (Systat Software GmbH, Erkrath, Germany) software was used for statistical analysis. After confirming normal distribution of all values (Kolmogorov-Smimov test), a two-way analysis of variance for repeated measurements was performed to detect potential differences within and between groups. In case of significant group differences, *post hoc *comparisons were made by using the Student-Newman-Keuls test. Bradykinin-induced vasoconstriction was expressed as the peak P_a _minus baseline P_a_. For all statistical tests, significance was assumed when *P *was less than 0.05. Data are presented as mean ± SEM.

## Results

### Systemic and pulmonary effects in healthy animals and healthy animals with TEA

Healthy animals (SHAM) exhibited stable HR and MAP, which were not affected by the use of TEA (SHAM + TEA). Leukocyte count was higher in healthy animals treated with TEA than in SHAM animals without TEA (*P *< 0.05). Arterial blood gases were similar between SHAM and SHAM + TEA animals (Tables 1 and 2).

**Table 1:** Systemic hemodynamics, respiration, hemoglobin, and biochemical markers of the different groups

Variable	SHAM	SHAM + TEA	CLP 6 h	CLP 6 h + TEA	CLP 24 h	CLP 24 h + TEA
HR (beats/min)	396 ± 20	412 ± 24	387 ± 23	412 ± 30	464 ± 16^#^*	442 ± 37
MAD (mm Hg)	139 ± 3	130 ± 9	129 ± 8	145 ± 5	133 ± 3	126 ± 6
RR (rate/minute)	107 ± 8	95 ± 9	94 ± 9	74 ± 6^#^	130 ± 7^#^*	115 ± 5
Lactate (mmol/dl)	0.95 (0.9/1.2)	1.1 (1.0/1.53)	1.25 (1.1/1.45)	1.1 (0.93/1.4)	2.3^#^* (1.83/3.1)	1.4^§^ (1.3/1.8)
Leukocytes (1,000/μl)	5,217 ± 309	7,184 ± 552^#^	3,868 ± 878	3,194 ± 771^#^	2,076 ± 343^#^*	1,752 ± 552^#^
Hb (g/dl)	15.6 ± 0.5	14.8 ± 0.2	15.3 ± 0.7	14.3 ± 1.2	14.9 ± 0.2	14.4 ± 1.0
MPO (μU/mg protein)	0.48 (0.42/0.57)	0.47 (0.42/0.81)	0.92 (0.87/1.08)^#^	0.97 (0.83/1.1)^#^	2.0 (1.91/2.17)^#^	0.26 (0.2/1.4)^§^
W/D	4 ± 1.4	4.4 ± 0.2	5.5 ± 0.2^#^	5.4 ± 0.3^#^	5.5 ± 0.2^#^	5.5 ± 0.1^#^

Measurements were performed in awake rats. HR = heart rate; MAD = mean arterial pressure; RR = respiratory rate. Data are expressed as mean ± SEM or median, 25%, and 75% percentile. ^#^P < 0.05 versus SHAM; †P < 0.05 versus all CLP groups, ^§^P < 0.05 versus same group without TEA; *P < 0.05 versus hyperdynamic sepsis (CLP 6 h).

**Table 2:** Arterial blood gas analysis of the different groups in awake rats spontaneously breathing room air

Variable	SHAM	SHAM + TEA	CLP 6 h	CLP 6 h + TEA	CLP 24 h	CLP 24 h + TEA
pH	7.44 ± 0.01	7.47 ± 0.02	7.42 ± 0	7.42 ± 0.01	7.44 ± 0	7.36 ± 0.03^#§^
PaCO_2_	38 ± 0	38 ± 0	40 ± 0	41 ± 2	37 ± 0	47 ± 2^#§^
PaO_2_	97 ± 2	92 ± 3	100 ± 2	100 ± 5	93 ± 2	86 ± 6^#^
HCO^3-^	24.8 ± 0.3	26.9 ± 1.2	25.3 ± 0.3	26.3 ± 0.9	25.8 ± 1.3	24.9 ± 1
ABE	1.0 ± 1	3.5 ± 1	1.3 ± 0	2.3 ± 1	1.5 ± 0.5	-0.4 ± 1.4

Data are expressed as median ± SEM. ^#^*P *< 0.05 versus SHAM; ^§^*P *< 0.05 versus same group without TEA. ABE = actual base excess.

**Figure 2:**
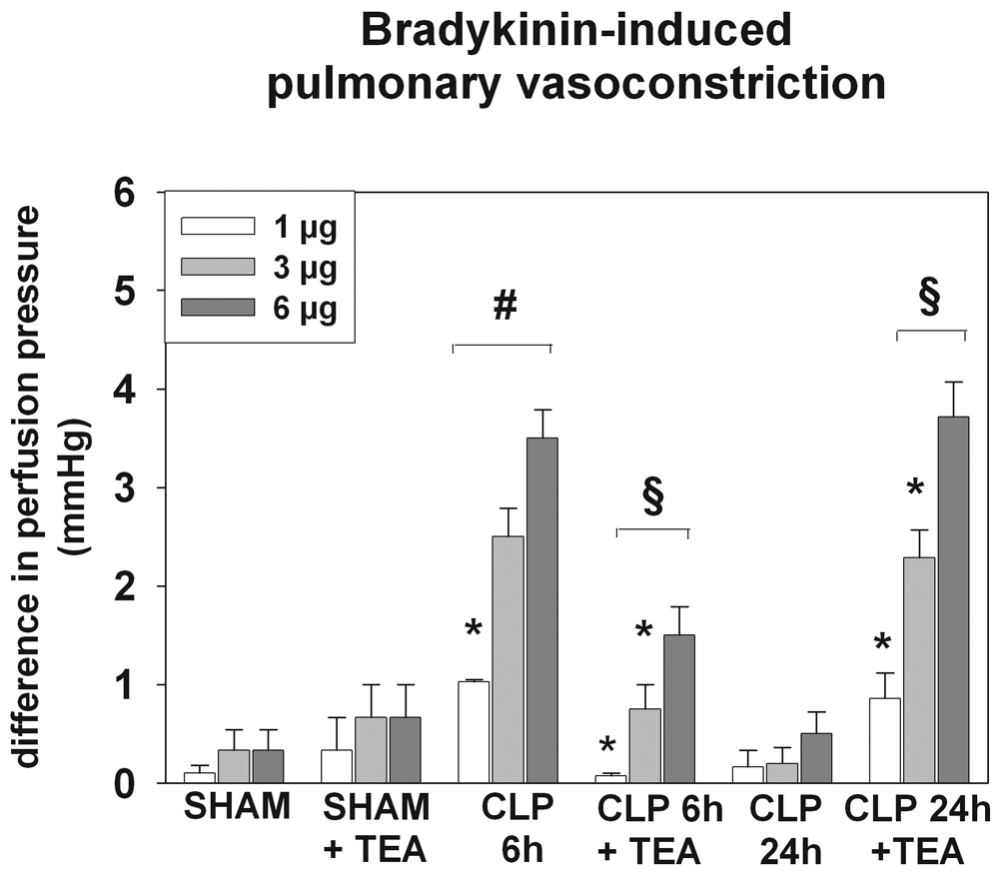
Exhaled NO (parts per billion) in sham animals, early and late sepsis, when compared with sham and septic animals treated with TEA (bupivacaine 0.5%; n = 6 in each group). ^#^P < 0.05 versus SHAM; ^§^P < 0.05 versus groups without TEA. Data are expressed as mean ± SEM.

**Figure 3:**
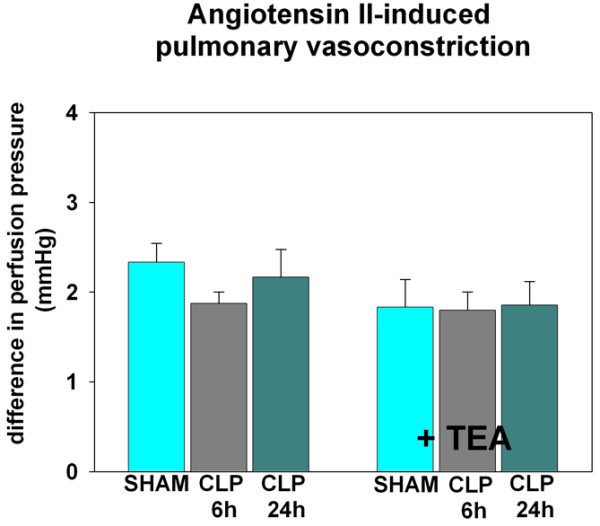
Comparison of bradykinin (BK)-induced vasoconstriction in isolated perfused rat lungs for evaluation of endothelial dysfunction (n = 6 in each group). The difference in perfusion pressure due to different concentrations of BK (1, 3, and 6 μg) is demonstrated. Data are presented as mean ± SEM. ^#^*P *< 0.05 versus SHAM; ^§^*P *< 0.05 versus the same group without TEA; * *P *< 0.05 versus BK, 6 μg, within group.

Pulmonary MPO activity and exNO levels did not differ between SHAM + TEA and SHAM (Figure 2 and Table 1). Pulmonary endothelial integrity was maintained as indicated by the absence of bradykinin-induced pulmonary vasoconstriction (Figure 3). In addition the pulmonary wet/dry-weight ratio and myeloperoxidase activity remained unchanged under the treatment with TEA (Table 1).

**Figure 4:**
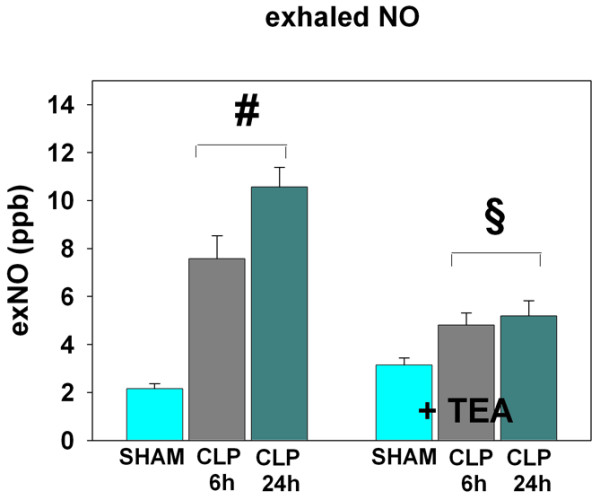
Angiotensin II-induced pulmonary vasoconstriction to test pulmonary smooth muscle cell function in healthy animals, early sepsis (CLP 6 h) and late sepsis (CLP 24 h) compared with groups receiving treatment with thoracic epidural anesthesia (TEA, n = 6 in each group). Data are expressed as mean ± SEM.

VSMC function in SHAM + TEA animals, as indicated by unchanged Ang II-induced pulmonary vasoconstriction was not different from that in SHAM (Figure 4).

### Systemic and pulmonary effects of TEA in hyperdynamic sepsis

In hyperdynamic sepsis (6 hours after injury), MAP and HR remained unchanged in untreated (CLP 6 h) and TEA-treated septic rats (CLP 6 h + TEA) when compared with SHAM (Table 1). CLP 6 h + TEA animals had a significantly lower respiratory rate than did hyperdynamic rats without treatment and SHAM animals (*P *< 0.05 versus CLP 6 h + TEA and SHAM; Table 1). Leukocyte count was significantly reduced in animals with sympathetic blockade when compared with the SHAM group (*P *< 0.05 versus SHAM; Table 1). No changes in pulmonary gas exchange were detected between the two hyperdynamic sepsis groups and SHAM animals (Table 2).

TEA prevented the increase of exNO concentrations in hyperdynamic sepsis, which was comparable to levels of healthy animals (*P *< 0.05 versus CLP 6 h; Figure 2). In contrast, MPO activity remained elevated, independent of sympathetic blockade (*P *< 0.05 versus SHAM; Table 1). The development of pulmonary endothelial dysfunction was attenuated under the treatment with TEA, as indicated by reduced bradykinin-induced pulmonary vasoconstriction (*P *< 0.05 versus CLP 6 h; Figure 3). Pulmonary edema remained increased in CLP 6 h and CLP 6 h + TEA (*P *< 0.05 versus SHAM; Table 1).

Ang II-induced pulmonary vasoconstriction remained unchanged in rats with hyperdynamic sepsis with and without sympathetic blockade and was comparable to those of SHAM animals (Figure 4).

### Systemic and pulmonary effects of TEA in hypodynamic sepsis

In hypodynamic sepsis (24 hours after injury), untreated animals (CLP 24 h) developed profound tachycardia, tachypnea, leukopenia, and increased serum-lactate levels when compared with healthy animals and with hyperdynamic sepsis (all *P *values <0.05 vs. SHAM and CLP 6 h; Table 1). Pulmonary gas exchange in the CLP 24 h group remained unchanged when compared with SHAM (Table 2). Under the treatment with TEA (CLP 24 h +TEA), hemodynamic variables did not differ from those in healthy animals, and serum-lactate levels were lower than those in untreated animals with hypodynamic sepsis (*P *< 0.05 versus CLP 24 h; Table 1). Arterial blood gas analysis revealed a decrease in PaO_2 _values when compared those in healthy animals (*P *< 0.05 versus SHAM) and an increase in paCO_2 _in TEA-treated rats when compared with CLP 24 h and SHAM (*P *< 0.05 versus SHAM and CLP 24 h; Table 2).

Sympathetic blockade reduced exNO and MPO activity below levels seen in the SHAM group (*P *< 0.05 versus CLP 24 h; Table 1 and Figure 2) but increased bradykinin-induced pulmonary vasoconstriction in contrast to the CLP 24 h group (*P *< 0.05 versus CLP 24 h and SHAM; Figure 3). Pulmonary edema was elevated in both treated and untreated hypodynamic sepsis (*P *< 0.05 versus SHAM; Table 1). Ang II-induced pulmonary vasoconstriction remained unchanged in TEA-treated and untreated hypodynamic sepsis and were comparable to the results of the SHAM group (Figure 4).

## Discussion

In the present study, the impact of a regional thoracic sympathetic blockade on pulmonary endothelial function was investigated in both hyperdynamic and hypodynamic sepsis by using an established rodent CLP model [11,13,17]. The major findings were (a) that implementation of TEA in hyperdynamic sepsis reduced pulmonary endothelial dysfunction, whereas sympathetic blockade in hypodynamic sepsis resulted in a deterioration of endothelial integrity, (b) that treatment with TEA exhibited antiinflammatory effects with modulation of the NO pathway in both hyperdynamic and hypodynamic sepsis, and (c) that TEA reduced neutrophil migration into the lungs only in hypodynamic sepsis.

One of the most established and clinically relevant sepsis models is the CLP procedure followed by fluid resuscitation and analgesia. It is a typical feature of this model that the time course of sepsis and the diversity of the bacterial flora closely mimic the clinical situation [9]. In this context, it is noteworthy that the CLP sepsis model is characterized by a biphasic hemodynamic response. Notably, an early hyperdynamic phase (2 to 10 hours after CLP) is followed by a hypodynamic circulatory phase between 10 and 20 hours [15,16]. Although several studies already addressed hemodynamic and intestinal effects of epidural anesthesia in CLP sepsis [14], it was still unclear whether (a) TEA has an impact on pulmonary endothelial (dys)function, and (b) the effects of sympathetic blockade on endothelial dysfunction differ in hyperdynamic and hypodynamic sepsis.

One established technique to investigate pulmonary endothelial dysfunction is the measurement of bradykinin-induced pulmonary vasoconstriction in isolated and perfused rat lungs [11,17]. In this context, Fischer and colleagues [11,17] demonstrated that administration of bradykinin into the pulmonary circuit results in a paradoxic vasoconstriction through BK_2 _receptors when the endothelial layer is injured after a septic insult [11,17]. In contrast, healthy control lungs with an intact endothelial layer lacked this bradykinin-induced vasoconstriction. In accordance with the observation of Fischer and associates [11,17,20], we also demonstrated that in hyperdynamic sepsis, administration of bradykinin resulted in a paradoxic pulmonary vasoconstriction in the current study. This effect was not seen in healthy animals and in healthy animals treated with TEA, indicating that animals of the two sham groups did not have endothelial injury. However, impairment of pulmonary VSMC function may have been responsible for a reduced vasoconstrictive response to bradykinin in the different groups. In this regard, it recently was reported that pulmonary VSMC function is affected at different stages of sepsis, thereby modulating pulmonary vasoconstriction [22]. Therefore, we decided to test VSCM function to exclude its influence by stimulating the receptor with Ang II as a different second messenger. With the observation of a uniform vasoconstrictive response in the different groups, it is unlikely that an impairment of VSMC function has contributed to the differences of bradykinin-induced pulmonary vasoconstriction among groups.

Pulmonary NO has been identified as a key mediator in the pathogenesis of endothelial dysfunction in both experimental sepsis and the clinical setting [17,23]. In animal sepsis models, selective inhibitors of the inducible NO synthase resulted in decreased exNO levels and significantly attenuated bradykinin-induced pulmonary vasoconstriction [17,19]. In this context, it is another finding of our study that TEA exhibited antiinflammatory properties by suppressing exNO as a surrogate marker of iNOS activity in both hyperdynamic and hypodynamic sepsis and additionally reduced neutrophil influx into the lungs in the hypodynamic phase. However, this antiinflammatory effect reduced endothelial dysfunction only in the hyperdynamic phase, whereas the impact of sympathetic blockade in hypodynamic sepsis promoted a deterioration of endothelial function. These results were somewhat unexpected, because iNOS generation from neutrophils and alveolar macrophages is known to play a critical role in the development of septic lung injury [6,24]. Moreover, Farley and colleagues [25] recently identified iNOS from alveolar macrophages as a key mediator in the development of pulmonary microvascular endothelial cell septic barrier dysfunction. One of the major roles of alveolar macrophages is to facilitate the recruitment of circulating neutrophils to the lungs, through production and release of neutrophil cytokines [26]. In addition, depletion of alveolar macrophages has been shown to attenuate septic neutrophil influx directly in murine septic lung injury [27]. Although not directly measured in our study, the present data suggest that TEA-related attenuation of MPO activity and reduction of exNO levels in the lungs may be dependent on changes of alveolar macrophage activity. This assumption is supported by a study of Fujii and colleagues [6], who demonstrated that inhibition of alveolar macrophages eliminated the increase in lung iNOS activity and protein expression and significantly attenuated the increase in pulmonary exNO product in endotoxemic rats.

The fact that TEA did not exhibit protective properties on endothelial integrity in hypodynamic sepsis despite its antiinflammatory action may be explained by a study of Traeger and colleagues [28], who reported that selective depletion of pulmonary alveolar macrophages increased lung injury in a peritonitis sepsis model by attenuating the defense mechanisms against gut-derived bacteria in the lungs. Our data are further supported by the observation of others that suppression of NO production reduces endothelial dysfunction in hyperdynamic sepsis [17]. However, this is the first study showing that the effects of sympathetic blockade time-dependently change their characteristics with the development of hypodynamic sepsis. In this regard, it is likely that TEA-related suppression of neutrophil migration and NO production reduced the host defense in the lungs, thereby deteriorating endothelial dysfunction [28]. Because we cannot exclude the possibility of negative cardiovascular effects under sympathetic blockade as a trigger for increased pulmonary endothelial dysfunction, future studies are needed to address this issue in more detail. This study has some limitations. First, the characteristic of this isolated perfused rat-lung model is an *ex vivo *investigation of pulmonary effects. Although this model is an established technique for studies of lung pathology [17,19,20,29] and allows measurements in the pulmonary circuit that are impossible in *in vivo *settings, further work is needed to confirm our findings *in vivo*. Second, we cannot exclude effects of systemic absorption of epidurally applied bupivacaine and subsequent antiinflammatory pulmonary effects. It was recently reported that intravenously administered local anesthetics accounted for reduced lung injury in different animals sepsis models [30,31], so further studies are needed to clarify the role of intravenously applied local anesthetics on inflammatory response in the presence of sepsis. Third, this experiment was not carried out to investigate the safety of TEA in critical illness, but to identify the effects of TEA on the pulmonary endothelial integrity. Although the use of TEA exhibited hemodynamic stability in hyperdynamic and hypodynamic sepsis, we are unable to comment on TEA-related actions on other organ systems. In this regard, however, it is noteworthy that the use of TEA in both hypodynamic and hyperdynamic endotoxemic sheep did not impair hemodynamics beyond the changes caused by endotoxin itself [32,33]. Although the use of TEA in critically ill patients has gained increasing interest in the recent years, its use in the intensive-care setting remains controversial [2-4]. Therefore, further studies are needed to assess the efficacy and safety of TEA in critical illness.

## Conclusions

In summary, this study demonstrates for the first time that TEA exerts protective effects on pulmonary endothelial integrity only in hyperdynamic sepsis. Whether the negative effects on endothelial function in hypodynamic sepsis have an impact on overall morbidity and mortality remains to be determined in future studies.

## Key messages

• Implementation of TEA reduced pulmonary endothelial dysfunction in hyperdynamic sepsis.

• Sympathetic blockade resulted in a deterioration of endothelial integrity in hypodynamic sepsis.

• Treatment with TEA exhibited antiinflammatory effects with modulation of the NO pathway in both hyperdynamic and hypodynamic sepsis.

• TEA reduced neutrophil migration into the lungs only in hypodynamic sepsis.

## Abbreviations

ABE: actual base excess; BK: bradykinin; CLP: cecal ligation and puncture; exNO: exhaled nitric oxide; iNOS: inducible nitric oxide synthase; MAC: minimum alveolar concentration; MPO: myeloperoxidase activity; NO: nitric oxide; OD: outer diameter; TEA: thoracic epidural anesthesia; VSMC: vascular smooth muscle cell.

## Competing interests

The authors declare that they have no competing interests.

## Authors' contributions

SL contributed to design, funding, data acquisition, and statistical analysis and drafted the manuscript; HF contributed to the design of the study and data acquisition; MW participated in data analysis and manuscript drafting; AZ participated in data acquisition and analysis; MF participated in data acquisition; HVA participated in the design and manuscript drafting; AWS contributed to planning the study and statistical analysis; LGF took part in study design, funding, data acquisition, statistical analysis, and drafting the manuscript.
